# Complete genome sequence of *Aquimarina muelleri* isolated from the shell of American lobster (*Homarus americanus*) with epizootic shell disease in Long Island Sound, New York

**DOI:** 10.1128/mra.00807-25

**Published:** 2025-09-12

**Authors:** Minseo Kim, Carlos Mejia Toro, Mehwish Ammad, Rachel Pak, Soo Jin Jeon

**Affiliations:** 1 Department of Microbiology, College of Bio-Convergence, Dankook University 477888, Cheonan, Republic of Korea; 2 Lewyt College of Veterinary Medicine, Long Island University 42805 , Brookville, New York, USA; Montana State University, Bozeman, Montana, USA

**Keywords:** *Aquimarina muelleri*, long read sequencing, American lobster, epizootic shell disease, whole-genome sequencing

## Abstract

We report the assembly and annotation of the complete genome sequence of *Aquimarina muelleri* isolated from the carapace of an American lobster with epizootic shell disease. The genome was sequenced using Pacific Biosciences technology and consists of a 5,320,888-bp chromosome with a G+C content of 31.52%.

## ANNOUNCEMENTS

*Aquimarina* is a Gram-negative bacterium belonging to the family *Flavobacteriaceae*. It is commonly found in the marine environment and has been reported to be highly predominant in shell lesions of American lobsters (*Homarus americanus*) affected by epizootic shell disease (ESD) (1, 2). However, the specific *Aquimarina* species involved and their associated virulence factors remain uncharacterized. Therefore, we isolated *Aquimarina* from lobster shell lesions and identified it at the species level as *Aquimarina muelleri*. Here, we present the complete genome sequence of *A. muelleri*, which will facilitate the identification and characterization of novel virulence factors involved in the pathogenesis of ESD.

The bacterial strain was originally recovered from a shell fragment of an American lobster (body length: 27 cm, weight: 741 g) harvested from Eastern Long Island Sound (41.18 N, 72.16 W). The shell fragment was stored in 15% glycerol at −80°C until use. To detach bacteria from the carapace surface, the fragment was thawed and aseptically incubated in 10  mL of marine broth at 37°C for 2  h, with vigorous vortexing three to four times during incubation. After enrichment, serial dilutions were prepared in marine broth and cultured on marine agar supplemented with kanamycin sulfate (100  µg/mL) and chitin (5%). Plates were incubated at 27°C for 72 h. Single colonies displaying orange-yellow pigmentation were selected and streaked onto fresh marine agar plates for purification, followed by incubation at 27°C for an additional 72 h. The colonies appeared rough and dry, exhibiting a sticky texture upon collection. Gram stain revealed long, rod-shaped Gram-negative bacteria. Genomic DNA was extracted from cultured colonies using the Quick-DNA Fungal/Bacterial Miniprep Kit (Zymo Research). The bacterial identity was initially assessed by real-time PCR (QuantStudio 3 System, Applied Biosystems) using the conserved 16S rRNA region within the genus *Aquimarina* and subsequently confirmed as *Aquimarina muelleri* by Sanger sequencing of the 16S rRNA gene using universal primers 27F and 1492R (Azenta).

**Fig 1:**
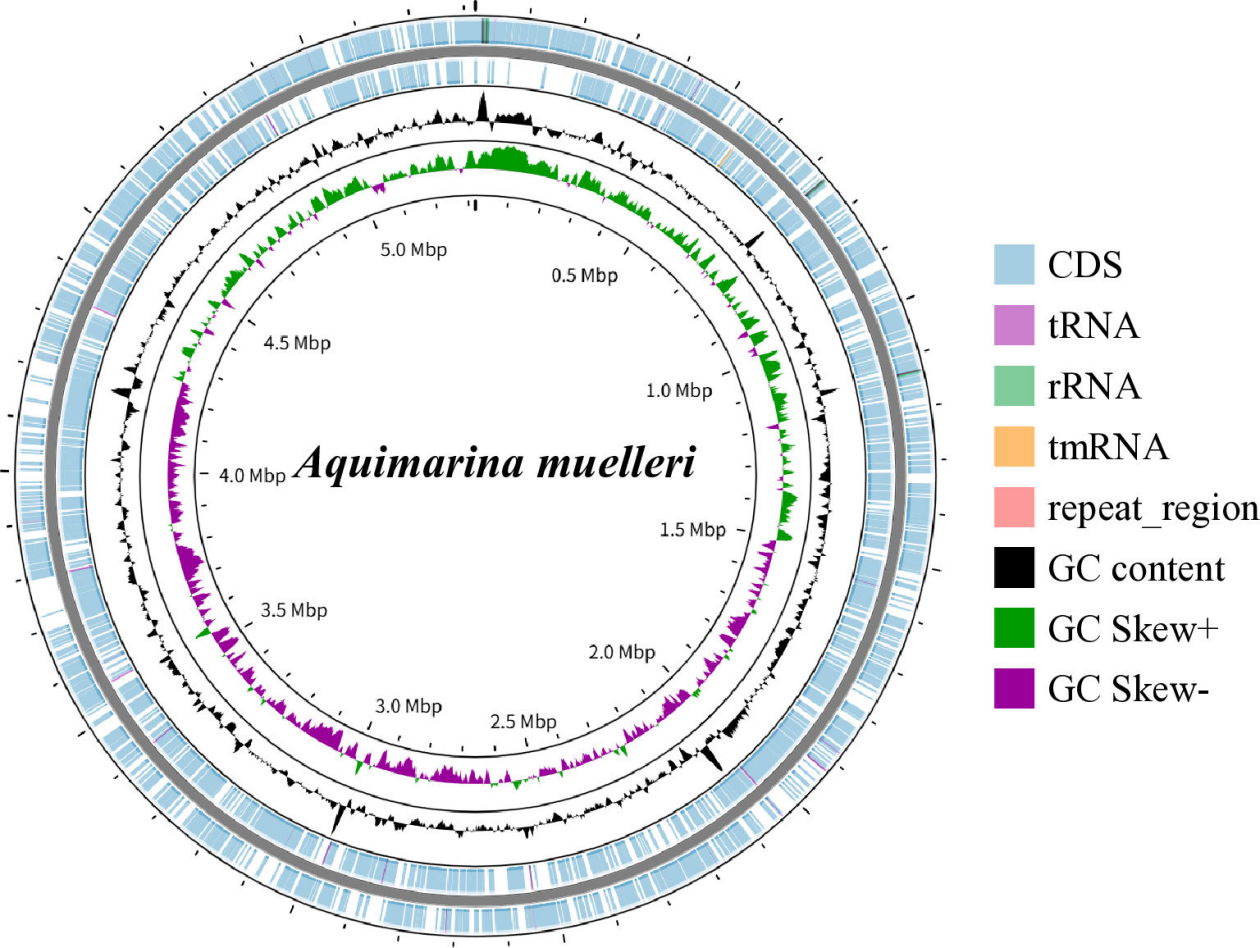
Circular genome map of *Aquimarina muelleri* isolated from the carapace of an American lobster with epizootic shell disease. The circular map was generated using Proksee (v.1.0.0a6).

The DNA was not sheared and was size-selected using BluePippen (size range: 9,000-50,000 bp). The genomic DNA library was constructed using SMRTbell Prep Kit 3.0 with the SMRTbell Adapter Plate 96A. Sequencing was performed using the PacBio Revio platform with SPRQ chemistry. The data were processed with PacBio SMRT Link v25.1. The analysis pipeline used lima for barcode demultiplexing and adapter removal, and HiFi circular consensus processing was applied to generate reads with a minimum quality score of QV20, generating 41,325 raw HiFi reads. The N50 of raw reads was 14,271 bp. The reads were assembled using Hifiasm (v.0.25.0-r726) (3), resulting in a complete genome of *A. muelleri* assembled into a single contig. Gene annotation was performed using Prokka (v.1.14.6) (4). Genome completeness was estimated using BUSCO (v.5.4.0) (5), and assembly quality was evaluated using QUAST (v.5.3.0) (6). The assembled genome was visualized as a circular map using Proksee (v.1.0.0a6) (7) and shown in Fig. 1. Default parameters were used except where otherwise noted. The assembled genome consists of a single contig of 5,320,888 bp with a genome coverage of 113×, a G+C content of 31.52%, and a completeness of 99.20%. The annotated genome includes 4,552 coding genes, 46 tRNAs, 12 rRNAs, and 1 tmRNA (Table 1).

**TABLE 1:** Assembly and genome features of *Aquimarina muelleri*

Description	Values
Genome size	5.3 Mb
Number of contigs	1
Contig N50	5.3 Mb
GC content (%)	31.52%
Completeness	99.20%
CDS	4,552
tRNA	46
rRNA	12
tmRNA	1

## Data Availability

This whole genome project has been deposited in GenBank under BioProject accession number PRJNA1293530 and BioSample accession number SAMN50024580. The raw read sequence is available in the Sequence Read Archive under accession number SRR34660033.
